# Drug-Delivery System Based on Salmon DNA Nano- and Micro-Scale Structures

**DOI:** 10.1038/s41598-017-09904-9

**Published:** 2017-08-29

**Authors:** Yunwoo Lee, Sreekantha Reddy Dugansani, So Hee Jeon, Soon Hyoung Hwang, Jae-Hyun Kim, Sung Ha Park, Jun-Ho Jeong

**Affiliations:** 1 Department of Nano Mechatronics, University of Science and Technology, 217, Gajeongbuk-ro, Yuseong-gu, Daejeon, 34113 Korea; 2 Nano-Mechanical Systems Research center, Korea Institute of Machinery and Materials (KIMM), 156, Gajeongbuk-ro, Yuseong-gu, Daejeon, 34113 Korea; 3 Department of Physics, Sungkyunkwan University, Suwon, 16419 Korea; 4 Research Institute of Advanced Materials (RIAM), Department of Materials Science and Engineering, Seoul National University, Daehak-Dong, Gwanak-Gu, Seoul, 151-744 Korea; 5 Department of Nano Mechanics, Nano-convergence Mechanical Systems Research Division, Korea Institute of Machinery and Materials (KIMM), 156, Gajeongbuk-ro, Yuseong-gu, Daejeon, 34113 Korea

## Abstract

Microneedles, fabricated by nano-moulding technology show great promise in the field of drug delivery by enabling the painless self-administration of drugs in a patient-friendly manner. In this study, double-stranded salmon DNA (SDNA) was used as both a drug-delivery vehicle and structural material with a microneedle system. SDNA is non-toxic and demonstrates good mechanical robustness, mouldability, biocompatibility, bio-absorbability, and binding affinity with drug molecules for bio-functional applications. Benign fabrication conditions to protect temperature-sensitive biomolecules are used to produce SDNA structures of various sizes with a high aspect ratio (4: 1). Unlike existing dissolving microneedle structure materials, the special binding characteristics of doxorubicin hydrochloride, anti-cancer drug molecules, and SDNA demonstrate the stability of drug-molecule encapsulation via UV-absorption and photoluminescence analyses. Based on COMSOL simulation and *in vitro* analysis of the stratum corneum of porcine skin, the mechanical functionality of SDNA microneedles was evaluated *in vitro* by penetrating the stratum corneum of porcine skin. The SDNA microneedle dissolved and drug permeation was assessed using rhodamine, a drug surrogate. Owing to its many beneficial characteristics, we anticipate that the SDNA microneedle platform will serve as an effective alternative for drug delivery.

## Introduction

The patterning of biological molecules, such as proteins, DNA, and cells, on surfaces has emerged as a useful mechanism to enhance the performance of bioelectronics^1–3^. Effective biomolecular patterns and structures are critical for producing effective microdevices. Accordingly, various fabrication techniques have been developed for biomolecular patterning to satisfy the requirements of biofunctional microdevices^2, 4^. In particular, microtransfer moulding has been utilized to fabricate patterns of biomolecules on designated substrates^5^. We hypothesized that this patterning technique could be combined with DNA to provide a useful new direction in the area of drug delivery.

Microneedles have come to the forefront owing to their great impact in the field of drug delivery. They readily and effectively enter the human body, without drug degradation, and serve as pain-free, micro-scale devices with easy operation. Traditional methods, e.g. hypodermic needles and oral drug delivery, induce pain or result in biomolecule degradation in the digestive system, respectively. Large biomolecules, like peptides, antibodies, and proteins, are strong candidates for the development of novel therapeutics, but their applications are limited, since the epidermis facilitates the permeation of molecules with a molecular weight of less than 500 Da^6–11^. Microneedles create small holes in the skin, through which a greater range of drug materials can be readily injected^12, 13^. Accordingly, microneedles have the potential to replace existing drug-delivery methods.

Three main microneedle types have been suggested: solid^14–16^, hollow^17, 18^, and dissolving microneedles^19–24^. Solid microneedles have associated safety issues; they can shatter in the skin, resulting in a biohazard. Hollow microneedles are limited by their complicated fabrication process and weak mechanical strength. In contrast, the tip and backing layer of dissolving microneedles dissolve, without leaving biohazardous substances during insertion, and the fabrication process is simple. Owing to these advantages, we focused on the development of a dissolving microneedle for drug delivery in this study.

However, issues remain in the implementation of dissolvable microneedles, including a lack of suitable materials to maintain their shape and limited stability under severe conditions, which restricts the kinds of drugs that can be delivered and the material costs. Overcoming these limits of transdermal delivery will enable microneedles to be utilized as a novel therapeutic mechanism^6, 7^.

To improve microneedle systems for drug delivery, materials that are mechanically robust^25^, biocompatible (i.e. do not damage protein integrity), and non-toxic (i.e. do not generate biohazardous waste) are needed. When penetrating the skin, the microneedle material strongly influences the insertion force required^19, 26–28^. Additionally, appropriate fabrication methods are needed to minimize damage at high temperatures.

Therefore, we utilized salmon sperm DNA (SDNA) as a microneedle application system; this material can generate mechanically strong, well-mouldable, biocompatible, bio-absorbable, and non-toxic devices. It can be extracted from the sperm of salmon using a cost-effective enzyme-isolation process. Double-stranded SDNA has the potential for use in biological, physical, and medical applications, e.g. in optoelectronic devices, biosensors, or drug delivery. DNA molecules confined on a solid surface for interactions with other molecules have received increasing interest^29–37^. Furthermore, enzymes or drugs could be blended with an SDNA solution because it has special binding properties that facilitate drug containment in microneedles^38^. These mixtures are mechanically strong and have high biocompatibility, surpassing the performance of other bio-materials used in microneedles, and remain stable in dried form for long periods^13^. Based on these characteristics, SDNA is an appropriate biomaterial for microneedle systems.

We expect that SDNA microneedles will be effective for drug delivery, including the delivery of anti-cancer therapeutics and vaccines. Owing to the beneficial properties of SDNA, SDNA microneedles can potentially be combined with other drug materials according to various therapeutic needs, including human growth hormone therapy^39, 40^, measles vaccines^41^, and influenza vaccines^42^.

In this study, we generated various three-dimensional DNA structures to verify the moulding characteristic of SDNA, ranging from a nano- to micro-scale. The suggested fabrication method is an aqueous-based, nano-moulding process that uses gentle techniques to allow the introduction of variably sized and high-aspect-ratio DNA structures and the protection of encapsulated temperature-sensitive drugs. During the fabrication process, we incorporated doxorubicin hydrochloride (DOX) into the SDNA microneedle and verified the binding characteristics. Furthermore, we performed several experiments to ensure that the SDNA structures have suitable mechanical properties for skin penetration for use as a microneedle. We built on the initial drug-delivery system using the three-dimensional DNA structure.

## Results

**Figure 1:**
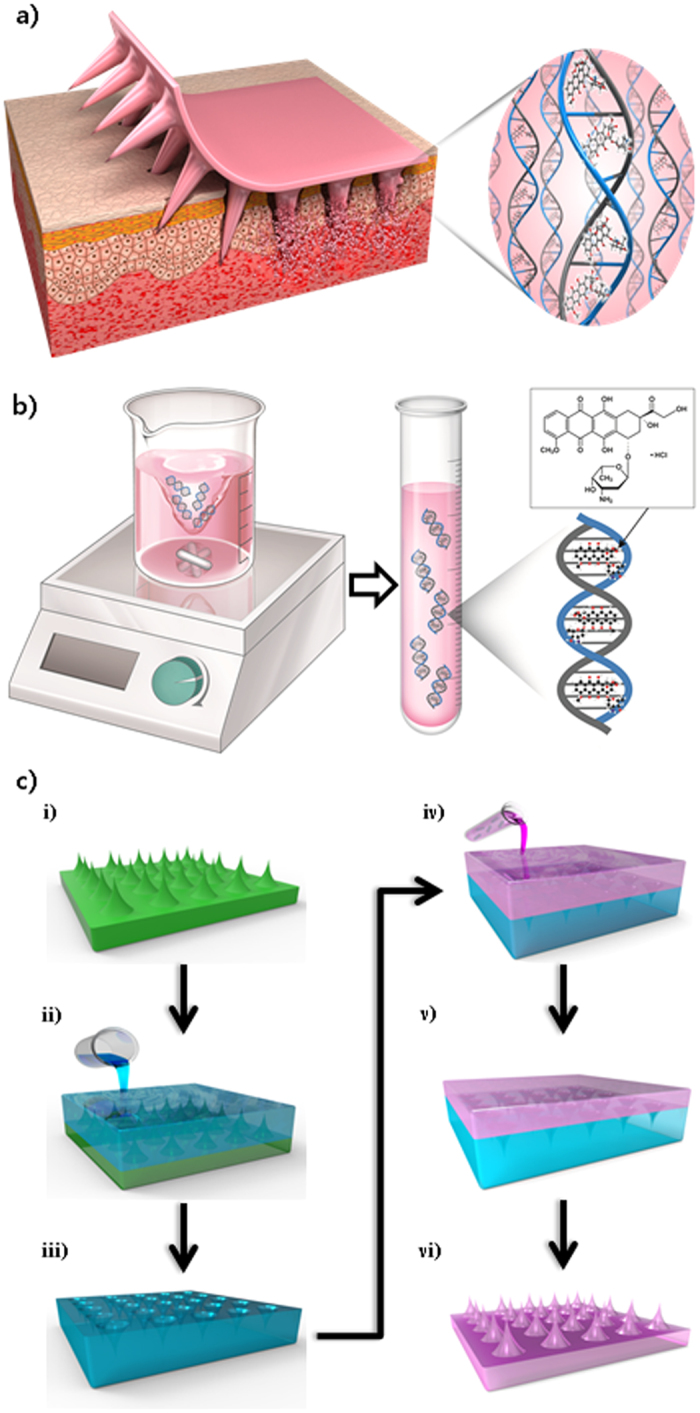
Schematic illustrations of the SDNA solution and structure fabrication, molecular structure of DOX, and DOX intercalation in SDNA. (**a**) Schematic diagram of drug delivery using the SDNA microneedle. (**b**) Generation of the SDNA solution with DOX by magnetic stirring, and a representative image of DOX intercalation into SDNA. (**c**) Schematic illustration of the fabrication process used to prepare an SDNA microneedle loaded with DOX. i) Microneedle array master. ii) PDMS is poured into the master to obtain the negative mould. iii) the PDMS mould is detached from the master. iv) the SDNA solution with DOX is poured into the PDMS mould. v) the SDNA solution with DOX is dried for more than 2 days. vi) the SDNA microneedle with DOX is detached from the PDMS mould.

Figure 1(a) demonstrates how the SDNA microneedles deliver anti-cancer therapeutics to the body. The DOX-doped SDNA microneedles penetrate the stratum corneum of the skin to make small holes for DOX dispersion. As the microneedle is inserted into the skin, the SDNA material is dissolved for DOX delivery. The DOX-doped SDNA solution was fabricated by magnetic stirring, as shown in Fig. 1(b). Following SDNA solution fabrication, both SDNA patterns and the microneedle could be fabricated, as demonstrated in Fig. 1(c). This method minimized damage to temperature-sensitive materials, since the mild fabrication process proceeds at ambient pressure and temperatures of less than 45 °C.

**Figure 2:**
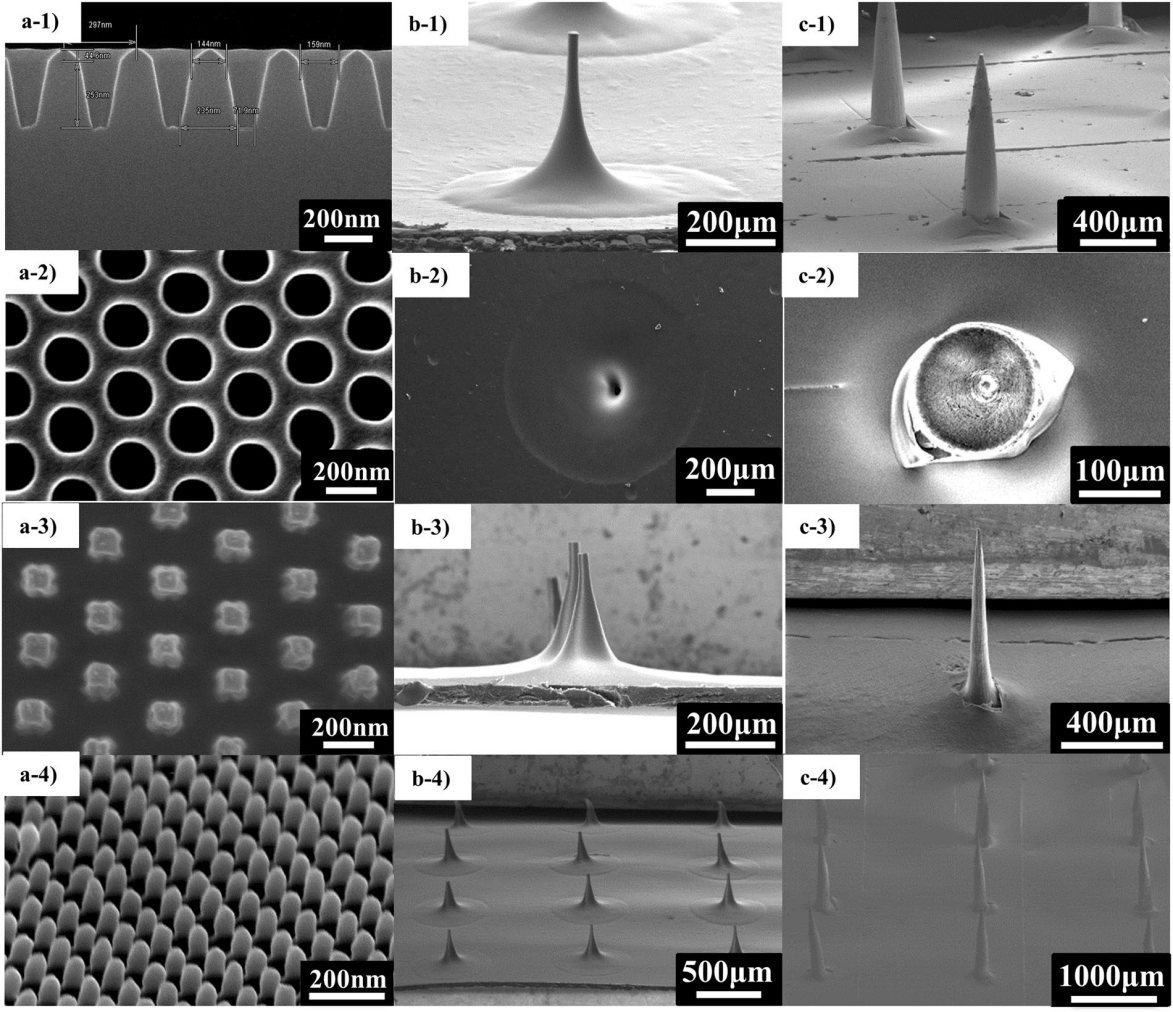
SEM images of the cylindrical hole pattern with a diameter of 150 nm and 300-nm spacing (300 nm in height), and images of the mould and DOX-doped SDNA microneedle patches. (**a**-1) Cross-sectional scanning electron microscopy (SEM) image of the master structure. (**a**-2) High-magnification SEM image of the second replica with UV resin. (**a**-3) High-magnification SEM image of the SDNA pattern. (**a**-4) Tilted SEM image of the SDNA pattern. (**b**-1) SEM image of the master template, 300 μm. (**b**-2) SEM image of the PDMS mould for the 300-μm microneedle. (**b**-3) SEM image of the SDNA microneedle (300 μm). (**b**-4) SEM image of the 3 × 3 array of the SDNA microneedle (300 μm). (**c**-1) SEM image of the master template (1000 μm). (**c**-2) SEM image of the PDMS mould for the 1000-μm microneedle. (**c**-3) SEM image of the single SDNA microneedle (1000 μm). (**c**-4) SEM image of the 3 × 3 array of the SDNA microneedle (1000 μm).

The nano-sized moulding process utilizes the excellent moulding characteristics of SDNA. First, a master structure was prepared by electron beam lithography with Si etching. Then, the master structure was moulded using ultraviolet (UV)-cured resin to create reverse moulds. This process facilitated the reproduction of numerous structures that could be reused. The UV-cured resin was chosen as a mould material owing to the rapid fabrication process and potentially high resolution of nano-scale moulding. The same process was repeated to construct the original patterns. These moulds were then utilized to prepare nano-sized structures. The SDNA solution was casted over the nano-sized moulds to fill cavities. After drying for more than 2 days at ambient pressure, without UV, at 45 °C, nano-scale DNA patterns were acquired, and these could be applied to biofunctional devices. As shown in Fig. 2a, 150-nm hole structures with a high aspect ratio (300 nm in height) could be easily obtained, indicating that SDNA has good mouldability. Based on these results, we could exploit SDNA to develop large structures with high aspect ratios for biological applications.

**Figure 3:**
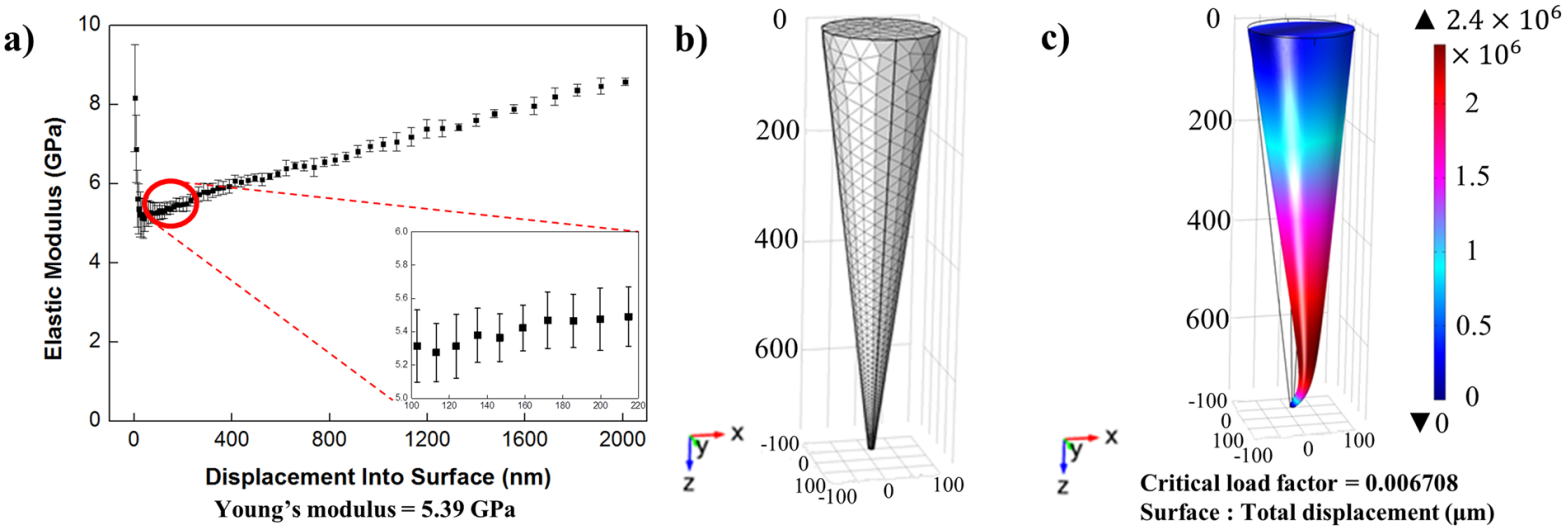
Mechanical-property simulation using COMSOL for SDNA microneedles when an axial load of 5 N was applied to the base of a single SDNA microneedle with a fixed constraint on the microneedle tip. (**a**) Young’s modulus of SDNA film. (**b**) Mesh analysis of the single SDNA microneedle. (**c**) Prediction of the buckling of the SDNA microneedle.

**Figure 4:**
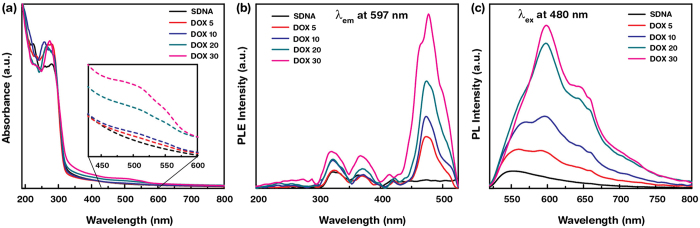
Absorption, photoluminescence excitation (PLE), and PL spectra of DOX-doped SDNA microneedles. (**a**) Absorption spectra of SDNA microneedles with various concentrations of DOX, i.e. 0, 5, 10, 20, and 30 μM (marked as SDNA, DOX 5, DOX 10, DOX 20, and DOX 30, respectively). (inset) For clarity, the absorption bands of DOX in SDNA microneedles in the wavelength range of 430–600 nm are magnified. (**b**) PLE spectra of DOX-doped SDNA microneedles at a fixed emission wavelength of λ_em_ = 597 nm. (**c**) PL spectra of DOX-doped SDNA microneedles at a fixed excitation wavelength, λ_ex_ = 480 nm.

These excellent moulding characteristics supported the use of SDNA to fabricate microneedles. We used commercial microneedles as master templates; they were 1000 and 300 µm high with a tip radius of approximately 20 µm, as shown in Figs 2b-1 and 2c-1. This master was utilized to fabricate a negative mould for microneedles with poly-dimethylsiloxane (PDMS) using a soft lithography technique (Fig. 2b-2, 2c-2)^43^. PDMS has a variety of useful properties, including a low surface energy, ease of use, conformal contact, low cost, and flexibility. Additionally, we altered the surface property from a hydrophobic to a hydrophilic nature by oxygen plasma treatment^44^. These properties of the soft elastomer material enable SDNA detachment from the microneedle with minimal damage, and the micro-sized structure can be readily reproduced^45^. Moreover, the porous structure helps to remove water from the elastomer^46^. Next, the SDNA solution was cast over the PDMS mould and solidified by drying for at least 2 days. During the process, shrinkage was observed in both the PDMS moulding and SDNA microneedles. The SDNA microneedle array was gently removed from the PDMS mould (Figs 2b-3, 4 and 2c-3, 4). We conducted numerous experiments to optimize the annealing time for fabrication. The benign temperature and aqueous-based fabrication process minimized damage to drugs and ensured a low cost so that it is competitive in the market.

We used SDNA as a microneedle material for several reasons. First, based on the Young’s modulus, SDNA is mechanically stronger than other individual dissolvable microneedle materials; therefore, it can maintain its shape when penetrating the skin^19^. Additionally, it can rapidly dissolve in water owing to the hydrophilicity of the sugar and phosphate molecules that make up the DNA backbone. Third, it has special binding properties, including intercalation between base-pairs, steric preference in major and minor grooves, and electrostatic interactions around the phosphate backbone. Desired drug materials can exhibit groove binding owing to their geometrical compatibility with designated base sequences and can intercalate with nucleobases via hydrogen bonding and π–π stacking^38, 47^. Finally, the SDNA solution is aqueous at room temperature, enabling fabrication at ambient pressures and under 45 °C to protect temperature-sensitive drugs.

For microneedles to replace existing hypodermic needle technology, they should exhibit sufficient strength to penetrate the stratum corneum. Ample mechanical strength for insertion is an important property for microneedle materials. In particular, Young’s modulus is closely related to the insertion force and buckling load of the microneedle^25^. We measured the Young’s modulus of SDNA, which ranged from 5.22–5.56 GPa (average, 5.39 $\pm$ 0.17 Gpa; n = 9), as shown in Fig. 3(a). The average value was larger than those of amylopectin (4.5 GPa)^48^, CMC (1 GPa)^19^, and PLA (5 GPa)^49^. Based on the Young’s modulus estimates, we conducted a COMSOL simulation to identify the extent to which the SDNA microneedle withstands a given axial load (Fig. 3). The critical buckling force of a microneedle can be estimated by multiplying the estimated critical load factor by the applied force. Based on the simulation, the critical buckling load for the SDNA microneedle was approximately 0.035 N, which is much larger than the force at which the needle can be inserted in the skin without buckling^50^. These results demonstrated that the SDNA microneedle has sufficient mechanical strength for practical applications.

UV−visible (Vis) absorption spectra were determined for DOX-doped SDNA microneedles, and the binding interactions between DOX and SDNA molecules were analysed. Figure 4(a) shows the absorption spectra as a function of wavelength for pristine and SDNA microneedles doped with various DOX concentrations (DOX; 0, 5, 10, 20, and 30 µM), referred to here as DOX 0, DOX 5, DOX 10, DOX 20, and DOX 30, respectively. The absorption spectra of DOX-doped SDNA microneedles showed characteristic absorption bands for DNA at wavelengths of ~260 nm and bands for DOX in the range of 470–550 nm. The DOX-doped SDNA microneedles showed increasing absorption above 400 nm with an increasing DOX, owing to the strong interaction between DNA and DOX *via* intercalation. For clear visualisation of the presence of DOX, a magnified graph is shown as an inset in Fig. 4(a). The intercalation of DOX into DNA molecules was probably due to strong π–π stacking interactions between the DNA bases and DOX molecules. Guanine and adenine are preferable for intercalation, whereas fewer interactions occur with thymine or cytosine^51, 52^.

Additionally, the photoluminescence (PL) characteristics of DOX-doped SDNA microneedles with respect to the DOX were analysed to determine the energy-transfer mechanism between DOX and SDNA microneedles and to provide support for DOX doping. PL requires proper excitation energy to transfer an electron from the singlet ground state to the excited state upon absorbing the energy of a photon. The inset of Fig. 4(b) indicates the PL excitation of the DOX-doped SDNA microneedles at a fixed emission wavelength (595 nm). An appreciable characteristic excitation peak was observed at ~480 nm and this excitation wavelength. PL emerges from the release of a photon upon relaxation of the electron from the triplet excited state. Because the absorption band of DNA molecules is in the range of 250 to 280 nm, energy transfer occurred by internal conversion within the excited singlet state, then from the excited singlet state to the triplet state via intersystem crossing, and finally to the emissive state^47, 53^. Figure 4(c) reveals the PL spectra of DOX-doped SDNA microneedles as a function of the DOX at a fixed excitation wavelength of 480 nm. These emission spectra showed three strong broad characteristic emission peaks for DOX at ~562 ~597, and ~650 nm, which were not observed for pristine SDNA microneedles. We noticed a change in the PL intensity as a function of the DOX, which revealed the degree of DOX binding to DNA molecules^54^. In addition, the emission peak area and full width at half maximum also increased as the DOX increased. This enhancement in PL (according to the DOX) was indicative of the degree to which DOX bound the DNA nanostructure in designated and improper regions, reflecting the size and shape of the DOX molecules. Significant changes in PL intensity for DOX-doped SDNA microneedles clearly revealed that DOX was loaded into the SDNA microneedles.

**Figure 5:**
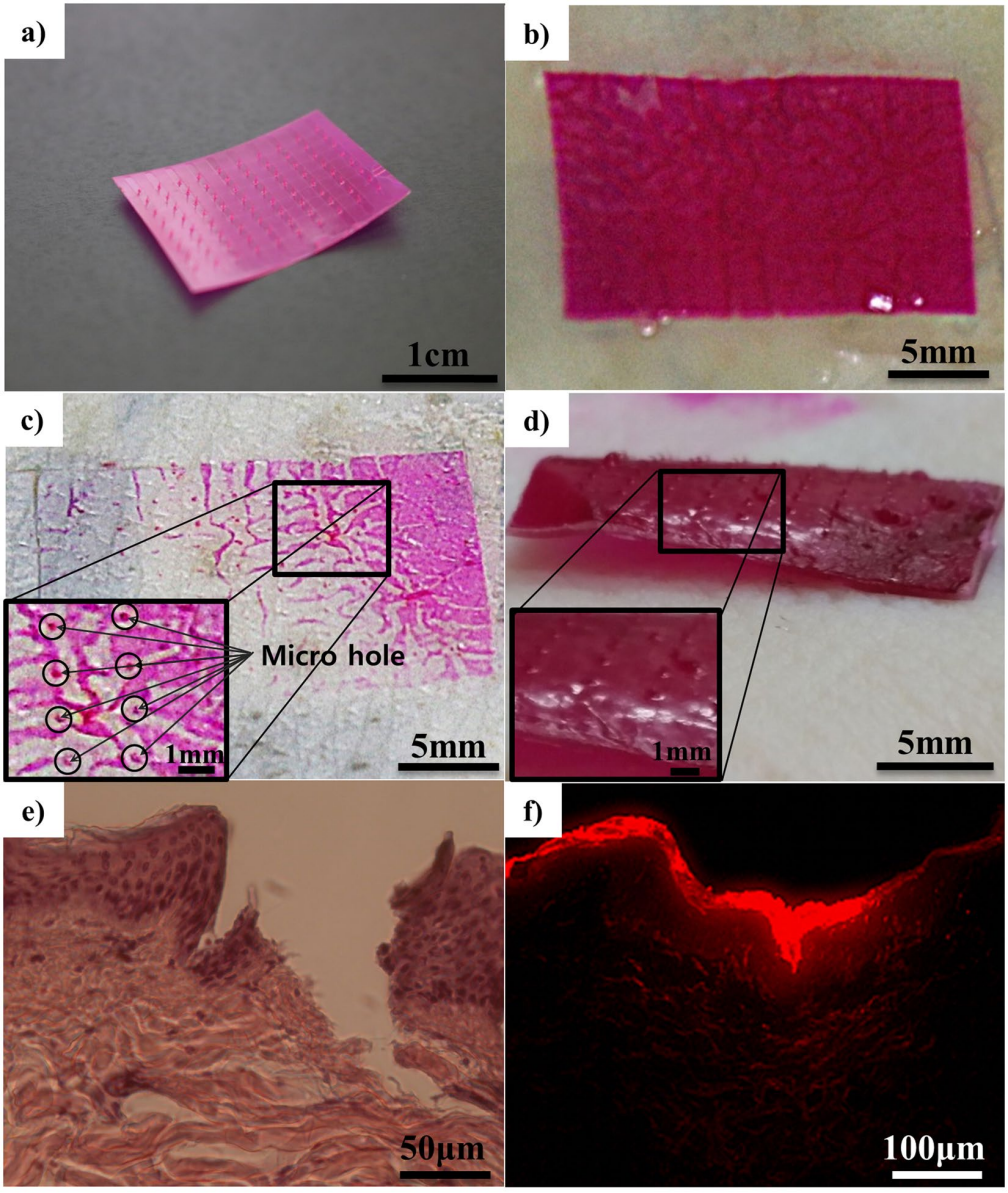
Microscopy images of SDNA microneedle insertion into porcine skin. (**a**) Image of a curled SDNA microneedle showing flexibility. (**b**) Application of the SDNA microneedle patch to porcine skin. (**c**) Microscopy image of porcine skin after removal of the microneedle patch. (**d**) Illustrative image of the dissolution of SDNA microneedle arrays after a 10-min insertion and subsequent removal from porcine skin. (**e**) Cross-sectional image of hematoxylin and eosin-stained skin at a SDNA microneedle-insertion site. (**f**) Fluorescence microscopy image of an SDNA microneedle with a drug surrogate.

To evaluate the practical application of SDNA microneedles (and confirm that they had sufficient mechanical strength and dissolvability), we inserted SDNA microneedles into porcine cadaver skin. Figure 5(a) shows a flexible SDNA microneedle patch loaded with DOX and fluorescent dye. Figure 5(b) shows a macroscopic image of the SDNA microneedle attached to the porcine skin. After a 10-min attachment to the porcine skin, the microneedles were detached from porcine skin and the insertion was traced by incorporating red dyes into the SDNA microneedle for visualisation (Fig. 5(c)). More than half of the microneedle dissolved within 10 min after insertion into porcine skin, as shown on Fig. 5(d). To visualise the mechanical functionality, the location of epidermal breach was verified by histology (Fig. 5(e)). The drug surrogate dispersed from the insertion site, supporting the application of the system for drug delivery (Fig. 5(f)). Based on the histological results, the depth of insertion was generally shorter than the height of the microneedles. This can be explained by the elastic nature of the skin and the geometry of the microneedles themselves, hindering their injection. Regardless of their mechanical characteristics, microneedles could be easily deformed at the surface of the skin during insertion. However, the SDNA microneedles had enough mechanical functionality to successfully penetrate the stratum corneum, and they dissolved completely, indicating their solubility (Supplementary Fig. S4)^16^.

## Discussion

Our results indicated that SDNA functions as both a drug-delivery vehicle and structural material for effective dissolving microneedles. SDNA microneedles represent a pragmatic and novel approach to directly deliver various drugs into the body with a non-toxic, bio-absorbable, and biocompatible structure material. This system could replace existing drug-delivery methods owing to its convenience, safety (as established by the Japan Foundation of Food Research Laboratory at Chitose for SDNA), and ease of use for delivering biomolecules into the human body. Owing to the good mouldability of SDNA, structures of various sizes ranging from the nano- to micro-scale at a 4:1 aspect ratio could be used to develop the microneedle structure.

Furthermore, the solution-based moulding process takes advantage of the special binding characteristics of SDNA, which enable strong chemical interactions to intercalate desired drugs, e.g. DOX, within the DNA structure. The unique intercalation characteristic of DNA molecules allows them to function as stable drug-delivery vehicles due to strong π–π stacking interactions.

Additionally, the SDNA microneedles demonstrated adequate mechanical functionality; the critical buckling load for the single SDNA microneedle was approximately 0.035 N. Using an *in vitro* model, the efficacy of the microneedle for the delivery of a drug surrogate over the stratum corneum of pigs was confirmed.

This novel DNA drug-delivery platform will be further developed for applications with various diseases; it overcomes the disadvantages of existing technologies for the delivery of a wide range of biomolecules, including vaccines, proteins, and peptides.

## Methods

### SDNA solution preparation

SDNA (1 g) was dissolved in 100 mL of de-ionized water to prepare the SDNA solution (Chitose Institute of Science and Technology, Hokkaido, Japan); the final concentration of the SDNA solution was 1.0 wt%. Magnetic stirring at approximately 1000 rpm for 10 h at room temperature was required to acquire a homogeneous mixture of SDNA and DOX or rhodamine^47^.

### SDNA doping with DOX

A suitable amount of DOX, i.e. 0, 10, or 30 μM, was pipetted into the 1.0 wt% SDNA solution. Then, the DOX-doped SDNA solution was prepared by vortexing for 5 min and incubation for 1 day at room temperature to acquire a homogeneous mixture^47^.

### Fabrication of a nano-sized mould

The first step in fabrication was the generation of a nano-patterned silicon master template, which was fabricated by electron beam lithography with Si etching. The dimensions of the arrays were 150 nm with a height of 300 nm (300 nm spacing). Subsequently, a UV-resin mould was used to fabricate the negative mould. UV resin was deposited on the surface of the master template and coated with a polyethylene phthalate (PET) film. Then, the back of the PET film was rolled with a hand roller to fill the cavities with UV resin. After 3 min of UV curing, the PET film was detached from the master template. The process was repeated to obtain the original mould.

### Microneedle mould fabrication

A commercial microneedle patch, the Acropass Microneedle Patch (Seoul, South Korea) and a commercial Derma Stamp (Larcobaleno, Seoul, South Korea) were utilized as a master stamp for the microneedle patch arrays. The microneedle patch arrays had heights of 300 μm (base width of 200 μm and tip radius of 10 μm) and 1000 μm (base width of 200 μm and tip radius of 10 μm). Subsequently, the female PDMS (Sylgard 184; Dow Corning, Midland, MI, USA) moulds were poured over the microneedle arrays to obtain a reverse polymer mould. The PDMS mould was manufactured by mixing the elastomer and curing agent at a ratio of 10: 1 by weight. The PDMS mould was then vacuum-treated for 1 h to remove air bubbles and cured at below 70 °C for 2 h. Subsequently, the PDMS mould was gently detached from the master stamp.

### SDNA pattern fabrication

The SDNA solution was cast over the UV-resin mould, which was attached to the substrate on a 2-inch Petri dish and dried in an oven (Thermostable ON-105; DAIHAN Scientific, Seoul, South Korea) at 45 °C for 2 days. After it solidified, the SDNA pattern was separated from the UV-resin mould.

### SDNA microneedle fabrication

Rhodamine B (>98% pure; Acros-Organics, Geel, Belgium), a water-soluble red fluorescent dye, was added at a concentration of 0.01 wt% to 1.0 wt% SDNA solution. Oxygen plasma treatment was conducted to modify the surface of the PDMS mould. The mixture was cast over the PDMS mould and dried in an oven (ThermoStable ON-105; DAIHAN Scientific) for 2 days. After it solidified, the SDNA microneedle patch was separated from the PDMS mould.

### Statistical analysis

Statistical comparisons of practical measurements of the heights of various structures were performed using one-way analysis of variance, and p < 0.05 was considered to indicate a statistically significant difference. Three different PDMS moulds were used, and 30 measurements were taken for each mould.

### Young’s modulus measurement

The mechanical properties of a DNA film were characterised using nanoindentation (NanoIndenter XP; MTS, Eden Prairie, MN, USA) with a Berkovich diamond indenter. The Oliver–Pharr method was utilized to analyse the nanoindentation data to determine the representative modulus values with respect to the indentation depth^55^. The surface stiffness of the DNA film was measured based on the continuous stiffness measurement unit obtained using the NanoIndenter XP, and Poison’s ratio of DNA was assumed to be 0.3. The measured modulus was 5.39 GPa, which reflected the average value over a range of indentation depths from 100 to 200 nm. The tests were repeated in nine trials, using the same samples.

### Simulation

The Structural Mechanics module of COMSOL Multiphysics software (www.comsol.com) was utilized for the simulations. The design of a single microneedle was a 3D cone-shaped structure of 750 μm in height, 200 μm in base diameter, and 10 μm in tip radius. The needle structure was considered a linear elastic material using the Young’s modulus, with an estimated Poisson’s ratio of 0.3. A fixed constraint was adopted such that the needle base only moved in the axial direction. The critical load factor of the microneedles was simulated while applying 5 N of axial loading. The microneedle tip was fixed in a position and the other end, i.e. the microneedle base, was allowed to move only in the axial direction.

### UV–Vis spectroscopy

A spectrophotometer (Cary 5 G; Varian, Palo Alto, CA, USA) was used to measure the optical absorbance of the DOX-doped SDNA microneedles in the visible and UV regions (wavelengths between 800 and 190 nm). The spectrophotometer was equipped with two light sources: a deuterium arc lamp (near-infrared and visible) and a quartz W–halogen lamp (UV). The instrument also had two detectors: a cooled PbS detector (near-infrared) and a photomultiplier tube (visible and UV). The spectrophotometer measures the frequency-dependent light intensity passing either through a vacuum or through the sample.

### PL measurements

PL and PL excitation spectra of DOX-doped SDNA microneedles were obtained at room temperature using a Xe-arc lamp-equipped fluorometer (FS-2; Scinco, Seoul, Korea) at 25 W. Excitation spectra were obtained at a fixed emission wavelength (597 nm), and emission spectra were measured by exciting samples at ~480 nm.

### Microneedle injection imaging

SDNA microneedle patches were applied to whole pig cadaver skin (a female Micro-pig® weighing 25 kg) with a thickness of 2.0 ± 0.2 mm (Medi Kinetics Micropig®; Pyeongtaek, Korea). The test was approved by the Institutional Animal Care and Use Committee. All skin samples were received and stored at –80 °C until tests were conducted. Microneedle penetration was traced after injecting the SNDA microneedle array into the porcine skin *in vitro*. After applying the microneedle patch to the skin for 10 min, the needle-exposed side of the skin was examined by microscopy to identify the sites of microneedle insertion.

To view the cross-sectional images of microneedle-insertion sites, the microneedle-treated porcine skin samples were fixed for 20 h with formaldehyde (10%) and subsequently embedded in paraffin wax. The sample was cut into 20-μm sections using a microtome, and the slices were stained with haematoxylin and eosin. Then, a histological analysis was performed by microscopy to identify the insertion site of the SDNA microneedle.

Other microneedle-treated porcine skin samples were cryosectioned and bisected in the *z*-direction (30 μm). The sections were visualized by microscopy.

## Electronic supplementary material

Supporting Information
