# Supplementary material for: Drug-Delivery System Based on Salmon DNA Nano- and Micro-Scale Structures
Source: Sci Rep. 2017 Aug 29;7:9724. doi: 10.1038/s41598-017-09904-9 (PMC5574990; doi:10.1038/s41598-017-09904-9)
Supplement: Supplementary file 1 — Supporting Information [file 41598_2017_9904_MOESM1_ESM.pdf]

Supplementary Information

Drug-Delivery System Based on Salmon DNA Nano-and Micro-Scale Structures

Yunwoo Lee, Sreekantha Reddy Dugansani, So Hee Jeon, Soon Hyoung Hwang, Jae-Hyun Kim, Sung Ha Park, and Jun-Ho Jeong

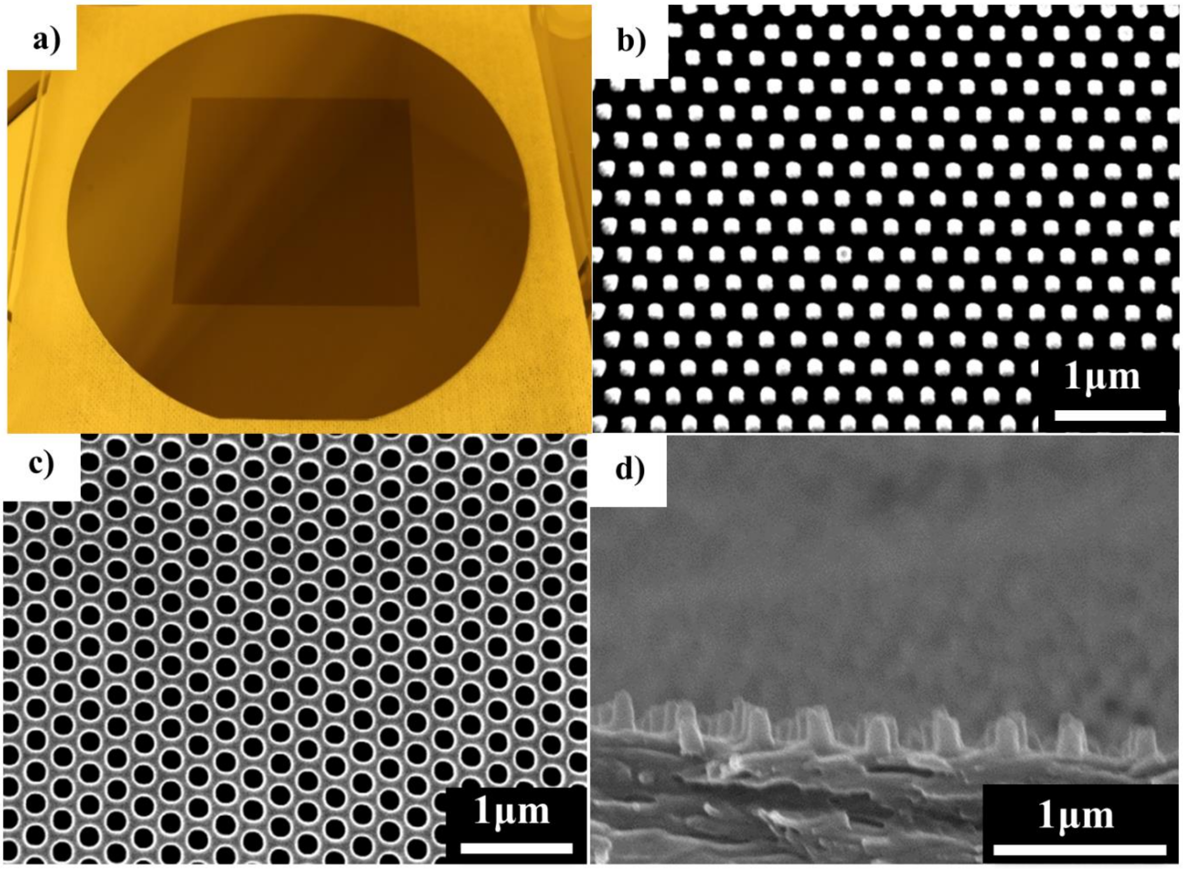

Figure S1| SEM images of the smoothing hole pattern of the master structure and the mould comprised of 150-nm diameter holes with a depth of 300 nm. a) Image of the master structure. b) SEM image of the first replica with UV-resin. c) SEM image of the second replica with UV-resin. d) Cross-sectional SEM image of the SDNA pattern

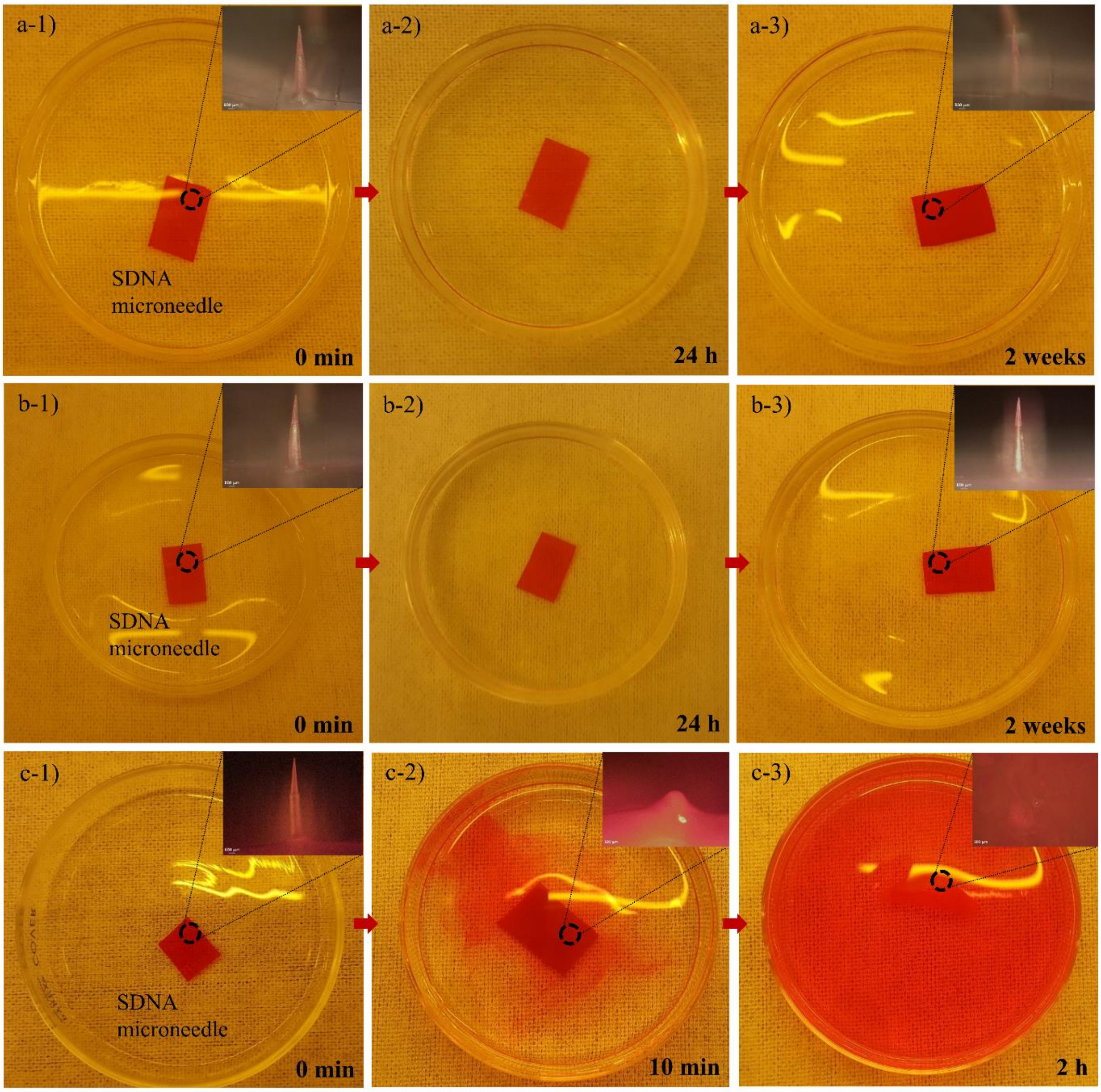

Figure S2| Optical images obtained at various stages of the chemical degradation of the SDNA microneedle patch and single microneedle (inset image) in ethanol (at room temperature), acetone (at room temperature), and PBS (at 37 °C). a-1) before immersion in acetone, and at a-2) 24 h and a-3) 2 weeks after immersion in acetone. b-1) before immersion in ethanol, and at b-2) 24 h and b-3) 2 weeks after immersion in ethanol. c-1) before immersion in PBS, and at c-2) 10 min and c-3) 2 h after immersion in PBS

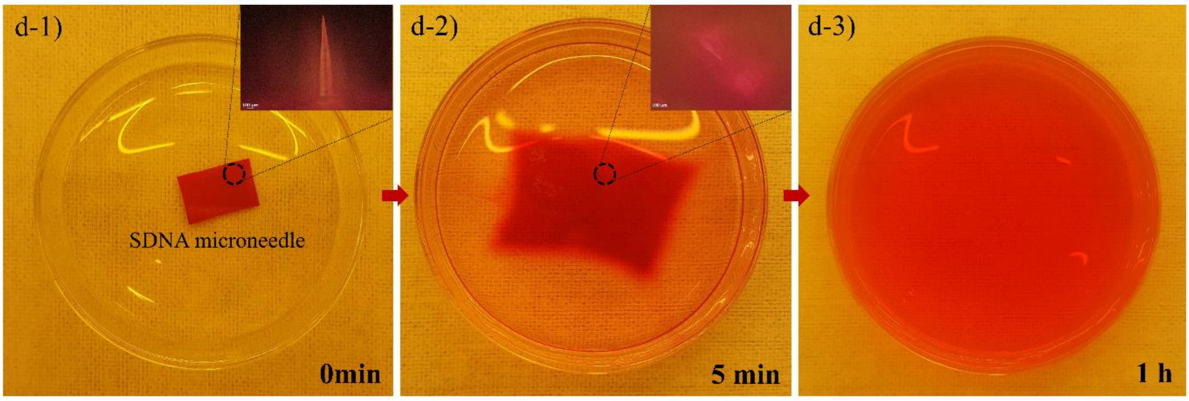

Figure S3| Optical images obtained at various stages during the dissolution of an SDNA microneedle patch in DI water at room temperature. d-1) before immersion. d-2) 5 min after immersion. d-3) 1 h after immersion

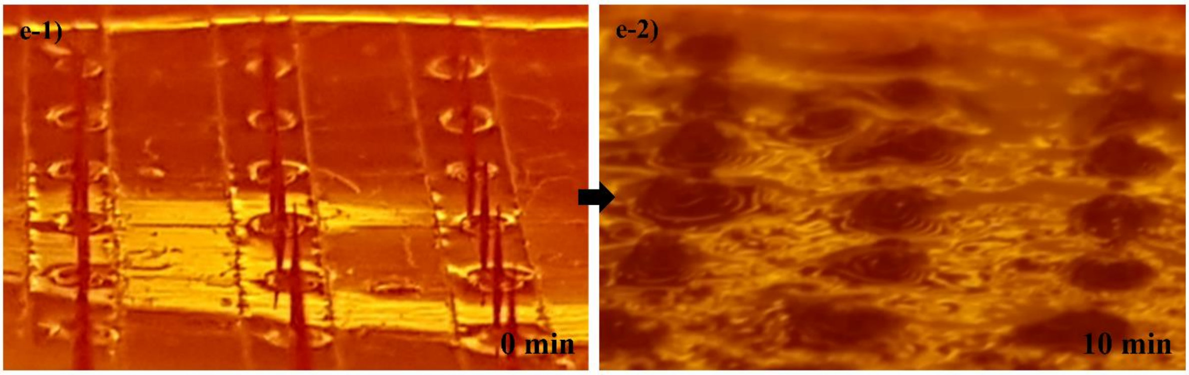

Figure S4| Optical images showing dissolution of the SDNA microneedle patch. e-1) before insertion into porcine cadaver skin and e-2) 10 min after insertion into porcine cadaver skin
